# Petrification in Contemporary Set Theory: The Multiverse and the Later Wittgenstein

**DOI:** 10.1515/krt-2023-0016

**Published:** 2024-11-05

**Authors:** José Antonio Pérez-Escobar, Colin Jakob Rittberg, Deniz Sarikaya

**Affiliations:** 16757 University of Geneva, Geneve, Switzerland; UNED, Madrid, Spain; Vrije Universiteit Brussel, Brussel, Belgium; Universität zu Lübeck, Lübeck, Germany

**Keywords:** multiverse, historicity, later Wittgenstein, petrification philosophy of mathematics, set theory, philosophy of mathematical practices

## Abstract

This paper has two aims. First, we argue that Wittgenstein’s notion of petrification can be used to explain phenomena in advanced mathematics, sometimes better than more popular views on mathematics, such as formalism, even though petrification usually suffers from a diet of examples of a very basic nature (in particular a focus on addition of small numbers). Second, we analyse current disagreements on the absolute undecidability of CH under the notion of petrification and hinge epistemology. We argue that in contemporary set theory the usage of construction techniques for set-theoretic models in which the Continuum Hypothesis holds and those in which it fails have petrified into the normative demand that CH remain undecidable. That is, the continuous and successful practices involving the construction of various set-theoretic models now act as a normative hinge shared among practitioners, i.e., have normative force in the discipline. However, not all hinges are universal, which is why we find disagreements in set theory. We will show that this is a refinement of, and partially conflicts with, the arguments presented by set theorist Joel David Hamkins.

## 1 Introduction

Stable natural phenomena have normative force for the natural sciences. Sustained experience with, say, objects falling to the ground puts a normative strain on empirical theories to explain these phenomena. Much the same has been argued for rudimentary mathematics. Ferreiros (2015), for example, argues that mathematical practices developed out of what he calls technical practices such as counting, measuring or drawing geometric shapes. Wittgenstein, in his *Lectures on the Foundations of Mathematics* (Wittgenstein 1976, hereon LFM) and *Remarks on the Foundations of Mathematics* (Wittgenstein 1978, hereon RFM), argued that this is true for mathematical practices more generally. He argued that sustained experiences with phenomena can take on normative force by a process he called *petrification*. His examples, however, remained at a rudimentary level of mathematics; e.g. he argued that our repeated experiences with counting two objects and then another two resulting in counting to four has petrified, leading to the normative force that “2 + 2 = 4” has today in that context. This, of course, is much in line with Ferreiros’ (2015) work. What is lacking, however, is an example of petrification in contemporary research mathematics. In principle, this is a challenge, because much of contemporary research mathematics is not connected to the empirical world as directly as in the case of simple arithmetic. Yet, in this paper we overcome this difficulty and provide an example of petrification in contemporary set theory. By doing this, we mainly aim to provide a contribution to Wittgensteinian scholarship. We also suggest a Wittgensteinian reading of Hamkins’ position that the set theorists’ *sustained experiences* with set-theoretic models in which the Continuum Hypothesis (from here on, CH) holds and those in which CH fails has petrified the undecidability of CH. This Wittgensteinian reading will partially align and partially conflict with Hamkins’ arguments and offers a reconstruction of Hamkins position that, we will argue, also contributes to its understanding. Basically, Wittgenstein’s view of mathematics accounts for the tolerance towards what would otherwise be considered “structural flaws” in mathematics, such as contradictions or, our focus in this piece the undecidability of CH.

Hamkins (2012) writes

As a result [of the set theorists’ experiences with CH and ¬CH models], I argue, the continuum hypothesis can no longer be settled in the manner formerly hoped for, namely, by the introduction of a new natural axiom candidate that decides it. Such a dream solution template, I argue, is impossible because of our extensive experience in the CH and ¬CH worlds.

The quote above is a striking example of a mathematician arguing for a mathematical position based on pre-existing practices (beyond the stipulation of axioms). However, why do pre-existing mathematical practices constrain further, contemporary research mathematics? The role of experience in deciding matters like the CH should be negligible under the most common views of mathematics, platonism and formalism. For a platonist, the deciding factor is the existence of mathematics in a realm of ideas, not our experience with such objects. For a formalist, truth flows from initially given axioms through logically valid steps to conclusions, which makes these conclusions impervious to any prior experiences with the given formal system. For both platonism and formalism, it is mathematical structure and not our experiences with mathematics what decides mathematical truth.

Still, the constraints of pre-existing practices have been noted by (Kant and Sarikaya 2021; Perez-Escobar and Sarikaya 2022), who argue that the process of mathematization does not end after simple real world observations. Instead, more abstract parts of mathematics are grounded in previously existing mathematical practices. Felix Klein also noted this, in a more general manner: for him, historical continuity is a basic regulatory principle in mathematics (Klein 2010, p. 7). If there was no such regulatory principle, how would a discipline that is mostly unconstrained by the empirical world display at least some degree of continuity? The Wittgensteinian notion of petrification, if it can be successfully applied to contemporary research mathematics, would constitute an explanation of this historical continuity. In this paper we argue that some ‘phenomena’ of set-theoretic practice have petrified in a Wittgensteinian sense, i.e., that they have taken on normative force. To work out what these ‘phenomena’ are and the implications of their petrification is a principal aim of this paper.

This paper is structured as follows. In section two we introduce Wittgentein’s notion of petrification in more detail. Section three gives a short presentation of the set-theoretic pluralism debate in which Hamkins puts forward his arguments. Section four then briefly presents Hamkins’ main points, and then moves to a Wittgensteinian reading of the situation in terms of petrification. In section five we return to the notion of petrification and flesh it out in more detail with the multiverse example at hand and we apply the petrification idea to other areas of set-theoretic activity. In section six we briefly discuss petrification in other areas of mathematics. Section seven offers a succinct conclusion and indicates how this work may be extended.

## 2 Wittgenstein and Petrification: An Alternative Regulative Principle to Platonism and Formalism in Mathematics

It has been argued that the later Wittgenstein’s philosophy of mathematics (LFM, RFM) offered insights to understand mathematics in practice. In recent years there has been a renewed interest in this direction (Bangu 2012; Berg 2024; Fairhurst, Pérez-Escobar, and Sarikaya 2024; Kusch 2016; Pérez-Escobar 2022, 2023a, 2023b; Pérez-Escobar and Sarikaya 2022; Pérez-Escobar and Sarikaya 2024; Steiner 2009; Wagner 2019; Zeng 2022). Many of Wittgenstein’s ideas were presented with a limited number of examples and need further work. We will argue that the notion of ‘petrification’ is helpful to understand Hamkins’ view on contemporary set theory.

The basics of the petrification idea are as follows. In daily life, we count in specific ways bound to social practices, and we often get the same results over and over. For instance, in our practices of counting discrete objects that do not merge together, we get that 2 + 2 = 4 almost always. We often get the answer 4 in cases of the addition of discrete objects. Our consistent experience with addition as a practice about ‘putting discrete things together’ makes it “petrify”, so that eventually it is not an ordinary empirical proposition, but a normative one: if at some point we counted only 3 objects, we would rather say that we lost one object, or we did not count well, or that our senses deceive us, but the mathematics is petrified and carries normative force.1Such a normative force sets Wittgenstein’s philosophy of arithmetic apart from empirical approaches like that of J. S. Mill: arithmetic is not ultimately empirical for Wittgenstein, as it regulates empirical evidence, for instance by discarding counterexamples or adjusting counting (see Pérez-Escobar 2023a). In this sense, a mathematical proposition like 2 + 2 = 4 turns from a statement verified by continued experience into a rule that may challenge future experiences. There are also cases where such an addition is simply not intended to work, like the often cited case of adding water droplets together, but this is a delimitation of the scope of the mathematical proposition, not its revision.

However, it is not clear how this idea relates to other mathematical practices, especially pure mathematics. Most of the literature on Wittgenstein’s notion of petrification has focused on simple applied cases like the above arithmetic (Bangu 2012), simple geometry (Pérez-Escobar 2022, 2023b; Zeng 2022) and mathematical modelling in the sciences (Pérez-Escobar 2023a) (the reader is referred to these sources for added details on the idea of petrification, although our recapitulation features the basics for our purposes in this paper). What happens in those cases that are too big to be counted in the manner above, or mathematics without such direct applications? This idea of petrification has been expanded by Steiner (2000, Section 4), who proposed that petrification occurs by calculation and proofs in general as well. These techniques are embedded in mathematical practices just like counting, and make mathematics resistant to revision. Interestingly, this happens in mathematics without direct applications. An example discussed by Steiner (2000) is finding the next prime number, and the conclusion that there is no last prime number. When presented with the task of finding the prime number that follows another prime number, most people perform certain calculations and get the same result. After the regularity that most people do get that result again and again, that result petrifies as the next prime in the succession. Another notion that petrifies is that people can always find a new prime after the last one. In other words, the conclusion that there is no last prime is a result of reasoning about these repeated experiences, despite being qualitatively different from the result of finding the next prime. Moreover, the proof that there is no last prime further petrifies the latter notion, further immunising it against doubt and potential counterevidence, and regulating subsequent mathematical practices. They key idea here is that the relevant empirical regularity for the petrification process is how practitioners themselves regularly perform mathematics (even in the absence of direct applications as in the case of apples),2Note, however, that a later Wittgensteinian reading of pure mathematics also admits applications in this area, although these are different from those of applied mathematics (Pérez-Escobar and Sarikaya 2022). and the additional contribution of proof to this process.3Further points in similar directions can be made about prime numbers. For instance, we often use a phrase to define them along the lines of “a number that can only be divided by itself and 1”. But what does this mean for the case of number 1 itself? A classical reading of the definition would suggest that 1 is indeed prime, but nowadays we do not want this since it would, among other things, render the prime factor theorem false, i.e. that each natural number can be uniquely (up to permutation) be described as the product of prime numbers. A solution is to modify the definition, for instance as “a number is prime if it has exactly two divisors” or to clarify that the “and” in the definition above implies that the divisors are two different divisors. This solution was added to the definition implicitly. Interestingly, before the definition fully petrified into its current form (that is, including the aforementioned addendum and thus excluding the number 1), there were competing definitions. For instance, Hardy used to include 1 as a prime number consistently in the first 6 editions of his *A Course of Pure Mathematics*. Later editions did not include 1 as a prime number at least in some places, but not all. Even then, some mathematicians disagreed. The matter gets even more complicated given the status of 1 as a number (e.g., it was not clearly established in Euclid). For a longer study consider f.i. Caldwell and Xiong (2012).


At this point, it is important to demarcate the later Wittgenstein’s philosophy of mathematics from formalism and platonism. This is important because the case from set theory that we discuss later cannot be solved by appealing to the inner structural properties of mathematics, and even more, it is a case of disagreement in mathematics. One could say that 67 is the prime number that follows 61 due to the structure of mathematics (regardless of whether platonism or formalism are adopted as metaphysical views), and thus, it is because of this structure that most people will get that 67 is the next prime (the ones who get another result are wrong). However, this is not what Wittgenstein’s later philosophy of mathematics is about: the normative power that mathematics acquires has nothing to do with an alleged structure put forward by formalism or platonism. This is further illustrated by Wittgenstein’s distinction between experiment and calculation in mathematics (for a recent exposition on this, see Berg 2024). The first time that a given mathematical community of people with similar training performs a mathematical derivation (or in Wittgensteinian terms, follows a mathematical rule), an experiment is being performed, and the result of the experiment is the mathematical result. If the command in particular was “find the next prime number after 61”, the relevant empirical evidence would be how most people follow the mathematical rules and what results are obtained. Since this is an experiment, there are no right or wrong results in advance: knowing the “right” result in advance would preclude the need for an experiment. Wittgenstein makes this point in the LFM when discussing the example of a multiplication (136 × 51)) with Turing:

Well, suppose 90 per cent do it all one way. I say, “This is now going to be the right result.” The experiment was to show what the most natural way is – which way most of them go. Now everybody is taught to do it – and now there is a right and wrong. Before there was not. (LFM, p. 94)

This is radically different from mathematical results being right or wrong by virtue of mathematical structure, and unlike formalism and platonism, is compatible with the historicity of mathematics as a regulative principle. Rule-following is underdetermined by rules themselves, and is further governed by psychological, social and cultural factors (Pérez-Escobar and Sarikaya 2024). Thus, rule-following has an empirical dimension. We conduct initial experiments and gain experience from them on what seems “natural” and successful in a given community. While we are acquiring experience with rule-following within our specific community in novel cases, there is no right and wrong in mathematics, but eventually, there is: our mathematical practices have become petrified. Unlike formalism and platonism, this leaves open the possibility that things could have been otherwise (due to, e.g., different training or psychological characteristics of rule-followers), thus accounting for the historicity of mathematics as a regulatory principle:4Note that the petrified products of mathematical practices do, in many cases, become part of the training of people in different mathematical communities, ensuring historical continuity and conditioning how rules would be followed by most people in subsequent new cases.

If we had all of us always calculated 12 × 12 = 143, then that would be correct - that would be the technique. (LFM, p. 97)

The above, together, constitutes a toolset for analysis that can help us better understand the historical continuity of mathematics in general, and Hamkins’ claims about the multiverse in particular. The takeaway message is that petrification is not just a phenomenon of arithmetical reasoning, an impression we might get from some readings of the later Wittgenstein’s philosophy of mathematics, but a more general phenomenon in mathematics, including pure mathematics, that renders mathematical beliefs immune or resistant to revision. This offers inductive grounds to assert that the notion of petrification may be useful to analyse aspects of practices in pure mathematics like resistance to the revision of opinions in set theory.

## 3 The Set-Theoretic Pluralism Debate

Set theorists routinely come into contact with the paucity of the formal machinery of set theory: set theorists routinely face the fact that first-order set theory does not decide every question; there are no formal and generally agreed upon means to decide if new axioms should be added to the currently accepted standard axioms ZFC and if new axioms should be added, there is no generally agreed upon method to decide which of the many possible new axioms should be added; etc. (see e.g. Džamonja and Kant 2019; Rittberg 2016) for accessible overviews). This has spawned a debate amongst some set theorists and philosophers of mathematics about the philosophical implications of this paucity: the so-called set-theoretic pluralism debate. This section presents this debate.

Today it is well understood that the currently accepted formal machinery of set theory is not strong enough to either prove or disprove all formal set-theoretic propositions. The currently accepted axioms of set theory, ZFC, together with current standards of proof, do not allow to either prove or disprove the Continuum Hypothesis, CH, for example. The set-theoretic jargon for this is that CH is independent of ZFC.

The independence of CH (from ZFC) was proven in two steps. Gödel presented a model, the so-called constructible universe L, in which both the ZFC axioms and CH hold. Cohen (1963, 1964) introduced the notion of forcing, which allows one to extend a given model in carefully controlled ways, and used it to formulate a model in which the ZFC axioms hold but CH fails. Gödel’s and Cohen’s models together prove that ZFC can neither prove nor disprove CH, i.e. CH is independent from ZFC.

Set theorists today know of many formal set-theoretic propositions that are independent from ZFC. The principal method to show the independence of a proposition P remains the same as for the independence proof of CH: build a model in which the ZFC axioms and P hold, and build another model in which the ZFC axioms hold but P fails. Forcing remains a principal method for building these models. There are, of course, other methods, like using the completeness property of first order logic, or working with inner models, or the direct constructions of models, like in the construction of L.

Some set theorists and philosophers of mathematics are having a debate about the philosophical implications of the independence results. *Monists* argue that independence reveals a paucity of the currently accepted formal machinery of set theory that ought to be overcome. One way to do so is to argue for new axioms that would settle relevant independence issues. For example, one might argue that the axiom stating that every set is constructible (in Gödel’s sense), V = L, should be accepted. This would settle the question whether CH is true: the axiom system ZFC + V = L proves CH. However, V = L is largely viewed as an unsuitable axiom-candidate to be added to the formal machinery of set theory; see (Maddy 1992) for references and arguments. This is indicative of the problem monists are currently facing. Those who wish to settle these issues by means of proofs from accepted axioms need to propose a new (set of) axiom candidate(s) and produce convincing arguments that these axioms should become part of the commonly accepted set-theoretic formal machinery.

*Pluralists* on the other hand argue that the paucity of the formal set-theoretic machinery reveals something about the nature of set-hood. A pluralist like Hamkins argues that it is not the paucity of these methods that needs to be overcome, but rather the idea that set-hood is a determinate notion. For such a radical pluralist, the question “is there a set with certain properties”, such as the Continuum Problem “is there a set bigger than the natural numbers but smaller than the real numbers?”, may not have a determinate answer. The pluralist embraces the idea that some mathematical questions simply cannot be answered in terms of true/false; the only answer we can give to such questions is “it depends (on the set-theoretic model under consideration)”.

We may further distinguish between epistemic and ontological forms of monism and pluralism in the set-theoretic pluralism debate.

The *epistemic monist* holds that, in principle, one can justify an answer to every (natural) set-theoretic question.5What makes a set-theoretic question ‘natural’ is beyond the scope of this paper. Obviously, Gödel’s results show that such justification may not be a formal proof from a given axiomatic system. Nonetheless, the epistemic monist holds, there are reasons (that ought to be generally accepted) that justify the answer to any (natural) set-theoretic question.

*Epistemic pluralists* hold that some set-theoretic questions cannot be justified by commonly accepted set-theoretic means. We just saw that Hamkins is an example of an epistemic pluralist: Hamkins believes that there is no justification to give a true/false answer to the Continuum Problem. The only answer we can give is ‘it depends on the model under consideration’. Epistemic pluralism forms a spectrum. Some may believe that all statements that are independent of ZFC have no determinate truth value, whilst others may hold that some strengthening of ZFC can be argued for but nothing else. The interested reader is referred to (Koellner and Woodin 2009).

*Ontological monists* hold that there is one, so-called ‘true’, universe of sets. In this universe, each set-theoretic statement is either true or false. Hence ontological and epistemic monism go well together and are often conceived as a ‘standard view’. Yet there are ontological monists who argue that, whilst in the true universe of sets every statement is either true or false, we (limited) humans may not have the means (now or ever) to come to know these truths. That is, ontological monism is compatible with epistemic pluralism: we might think that there is a fact of the matter about the set-theoretic universe, but we may lack justificatory means to get at those.

*Ontological pluralists* hold that there is no true universe of sets but rather many different set-theoretic universes. Together, these form the so-called ‘multiverse’. Which universes are part of this multiverse depends on the flavour of ontological pluralism under consideration.6Hamkins collects all set-theoretic models in his multiverse. Woodin and Steel collect only forcing extensions of some ground models in their generic multiverse (but they differ in the details which forcing extensions are collected in their respective multiverses). Ontological and epistemic pluralism go well together. Ontological pluralism does not seem to be compatible with epistemic monism, as the models need to disagree about at least some set theoretic statements (yet it might be possible that these models agree on all relevant statements, whatever we mean exactly with relevant statements (cf. Arrigoni and Friedman 2013)).

In this paper we are primarily interested in the petrification of experience into rules. This plays out on the epistemological dimension. We are therefore primarily interested in the epistemic dimensions of the set-theoretic pluralism debate in this paper. Hence, from now on any reference to monism/pluralism without epistemic/ontological specification is to be understood as epistemic monism/epistemic pluralism.

## 4 Hamkins’ Multiverse View and Petrification in Set-Theoretic Practice

In this paper we are particularly concerned with the arguments set theorist Joel David Hamkins has put forward in the set-theoretic pluralism debate. Hamkins has offered the perhaps most radical pluralist proposal to date. We first summarise his position in this section and then discuss petrification in the context of the set-theoretic pluralism debate.

Hamkins self-identifies as a Platonist.7He himself notes the similarities to Balaguer’s (1998) notion of full-blooded platonism. Sometimes the term plenitudinous platonism is also used. He is not (principally) concerned with classical problems of mathematical platonism, such as Benacerraf’s question how we might have access to abstract mathematical objects. Rather, Hamkins’ writing focuses on the ontological status of (some) mathematical objects, in particular on the ontological status of the various models of set theory that set theorists have produced.

According to Hamkins, the (ontological) monist holds that there is one model of set theory that correctly captures the notion of set. This model is commonly called the “true universe of sets V”. This true universe is the intended structure of set theory, the (ontological) monists claim. However, this structure has not been individuated yet. There are many candidate structures that might be the true universe of sets. It is the job of the (ontological) monist to produce convincing arguments that one of these candidate structures in fact captures the intended notion of set-hood.

Hamkins’ pluralism8Hamkins is both an epistemic and an ontological pluralist. As mentioned, we are particularly interested in his epistemic pluralism in this paper. is based on the claim that the set theorists’ experiences with the various models of set theory make it impossible to assign a special ontological status to one of them as “the true universe of sets”. Since no single universe is special in this sense, Hamkins regards them all as ontologically equal. That is, to understand the notion of set-hood, says Hamkins, is to understand how set-hood is explicated in the (literally infinitely many different) models of set theory.

Of particular interest to the purposes of this paper is how Hamkins motivates his pluralism in regard to CH. He writes:

I argue [that] the continuum hypothesis can no longer be settled in the manner formerly hoped for, namely, by the introduction of a new natural axiom candidate that decides it. Such a dream solution template, I argue, is impossible because of our extensive experience in the CH and ¬CH worlds. (Hamkins 2012)

He elaborates:

I claim now that this dream solution [i.e. introduce a new natural axiom candidate that decides CH] has become impossible. It will never be realized. The reason has to do with our rich experience in set-theoretic worlds having CH and others having ¬CH. Our situation, after all, is not merely that CH is formally independent and we have no additional knowledge about whether it is true or not. Rather, we have an informed, deep understanding of the CH and ¬CH worlds and of how to build them from each other. Set theorists today grew up in these worlds, and they have flicked the CH light switch many times in order to achieve various set-theoretic effects. Consequently, if you were to present a principle φ and prove that it implies ¬CH, say, then we can no longer see φ as obviously true, since to do so would negate our experiences in the set-theoretic worlds having CH. Similarly, if φ were proved to imply CH, then we would not see φ as obviously true, since this would negate our experiences in the worlds having ¬CH. The situation would be like having a purported ’obviously true’ principle that implied that midtown Manhattan doesn’t exist. But I know it exists; I live there. Please come visit!

The attentive reader will have spotted Hamkins’ use of the experience terminology. Hamkins suggests that sustained experience, or ‘rich experience’ as he puts it, with certain formal mathematical results that neither prove nor disprove a formal mathematical statement (CH in Hamkins’ case) can nonetheless have an impact on the truth-value of that statement (rather than just impacting our beliefs or justifications in this matter: Hamkins’ point is that CH is settled, not that one must justify an affirmative or negative solution, or that he believes that it should not be settled; in other words, he is not making an epistemic point). Thus, formalism, a very common view of mathematics which is especially relevant in foundational issues, is insufficient to account for what is going on in practice. Furthermore, in formalism, mathematics is predetermined by axioms before any experience with a concrete mathematical corpus, and thus, an appeal to experience is meaningless. Yet, as we suggest in this paper, this looks a lot like the Wittgensteinian idea of petrification: sustained observation of some regularity petrifies into a normative demand. Yet this raises the question why Hamkins (and presumably other pluralists) feel the force of this normative demand whilst monists (in general) do not seem compelled by it. Our discussion now turns from a presentation of Hamkins’ view to a Wittgensteinian discussion of petrification in contemporary set-theoretic practice.

Petrification happens to ways of doing things that produce some form of stable phenomena. Above we saw the example of counting two objects and then another two and routinely arriving at four objects. If this way of doing things is pragmatically successful, then it might petrify and take on normative force. If our counting of two and another two objects does not lead to counting four objects, the normative force of the petrified way of doing things compels us to believe that we have miscounted. Frege felt this normative force when he argued that empirical observation cannot undermine mathematical facts. From a Wittgensteinian perspective this is correct, but only the second half of the story. The first half is that there is first a way of doing things, and whether this way of doing things happens regularly and is successful is a pragmatic, and hence partially empirical, question. Furthermore, as we saw before, petrification also happens for empirical regularities whose pragmatics are less obvious, i.e., when there are no direct applications: mathematics has followed a given path because that is how mathematical rules have been followed by a given community in a given context (empirical component), and those practices have been insightful and valuable in other ways (pragmatic component). Once the dust settles things may petrify into the normative force that Frege was concerned with.

Hamkins highlights that there is a way of doing set theory today that involves the building and handling of various set-theoretic models; “Set theorists build models to order” (Hamkins 2012, 3). To this empirical observation about set-theoretic practice (ontological and epistemic) monists do not disagree. Neither do they seem to disagree that this is a successful way of doing set theory; these monists do not argue that set theorists should stop building set-theoretic models. In fact, they routinely point out that all these models can be conceived as submodels of the true universe of sets. That is, monists agree that the way set theory is currently done is successful and aim at keeping this successful practice.

We take from this that the practice, in its pre-theoretical understanding as ‘way of doing things’, of building various set-theoretic models has petrified into the normative claim that set theory ought to be such that these models can be built and studied.

Besides the above ‘way of doing things’, Hamkins makes an additional and more controversial claim, namely that all these models should be considered as metaphysical equals. To this the monists disagree. They insist that the experience with the various set-theoretic models Hamkins talks about does not have the kind of normative force that forces set theorists to give up (epistemic) monism (yet?). Hamkins argues that these experiences have, to put it in our terminology, petrified into the normative claim that CH is absolutely undecidable.9The undecidability of a proposition is a formal result. Absolute undecidability of a proposition is the philosophical view that the proposition can never be decided by any means (Gödel 1995; Väänänen 2014), see also (Rittberg 2021, 2024) Yet, not everybody agrees. Practices are heterogeneous, and the above experiences do not play the same role across communities or even individual practitioners. What petrifies for one may not petrify for another, i.e., petrification is sometimes a local phenomenon. This means that the experiences Hamkins relies on may have petrified for some set theorists into the normative claim that CH is, indeed, absolutely undecidable, yet not for others. The next section will engage with this heterogeneity in petrification via the notion of *hinges*.

## 5 Local Petrification in Set-Theoretic Practices

It seems that we need to take an extra step to further understand the petrification of the absolute undecidability of CH: regular practices and experience, and relevant proofs, did not completely petrify it. They petrified the independence of ZFC but this could constitute motivation to move beyond ZFC to reach a communal decision on CH. First, it is not a minor, somewhat ignored problem: CH is of interest to the set-theoretic community at large.I It was an open problem proposed by Cantor, reverberated by Hilbert, and there is an ongoing debate amongst some of the leading set theorists of our time about the Continuum Problem (we say “was”, since Hamkins’ point is that it is no longer a problem). Thus, its irresolution is not the consequence of a lack of interest. Second, the problem is about *prima facie* concrete mathematical objects, and therefore a decision should be in principle manageable. If you believe that there is one intended structure of set theory and have some reasonable assumptions, such as satisfying ZFC, then the CH has a determinate truth value in this structure. Due to these factors, the proof on the undecidability of CH from ZFC only resulted in a partial petrification. And indeed at least three intertwined programs started to try to settle the truth value of CH. First, the inner model program in which the idea is to find “L-like” models, which are not as restrictive as L. Second, a search for the correct axioms of infinity (or large cardinals as we would say today). Third, the use of so-called forcing axioms. This means there are suggestions to extend the axiomatic system ZFC. So the result can be read rather as a statement about ZFC, namely that there is a paucity to ZFC set theory.

To understand the petrification of the absolute undecidability of CH we need to stress the following: Hamkins capitalises on *experience* with *practices* involving models in which either CH or ¬CH holds, and our *experience* with the forcing-technique in particular that CH is a ‘switch’, i.e. a statement such that every model can be extended to one where the truth value of CH is different from the one we started with (every model of CH has a forcing extension where CH fails, and vice versa). Note how the terms coincide with the ones we used to describe Wittgenstein’s notion of petrification: a *practice* is carried out consistently, and repeated *experience* with it makes it petrify. In the case of the undecidability of CH, we get CH and ¬CH models, transitions between them, and Hamkins’ recurring *experience* (the fact that he explicitly alludes to experience further supports this narrative). Although this is not about counting, calculating or finding the next prime like in the simple sense in which we illustrated petrification, the key elements of this process are still present. Overall, what we have is a synergy of Wittgenstein’s original account of petrification in arithmetic and further elaborations for pure mathematics without direct applications (Pérez-Escobar and Sarikaya 2022; Steiner 2000) working towards the petrification of the absolute undecidability of CH, a synergy of *experience* and *proof* as part of more abstract set theoretical *practices*.

Of note, Wittgenstein was a harsh critic of “metaphysical talk” by mathematicians, which he called “prose” (see Dawson 2014; Maddy 1993; Pérez-Escobar and Sarikaya 2022). It is therefore unclear how metaphysical views would petrify in a Wittgensteinian sense, even under the extended account offered before. This does not mean that it would not be possible (as, for instance, metaphysical views could in principle be part of the training of mathematicians), but its characterization is outside the scope of this paper. Yet, fortunately for us, there is a correlate that is more distinctively mathematical and which can petrify unproblematically according to the account above: forbidding the inference of decidability. In fact, this is similar to physicists who, due to their local training, forbid the use of Dirac’s delta functions outside integrands (not doing so could lead to contradictions), despite being an otherwise legitimate mathematical procedure (this case has been analysed from a Wittgensteinian perspective too; see Pérez-Escobar and Sarikaya 2022, section 3.3).

Later on, in *On Certainty *(Wittgenstein 1969), his work on hinge epistemology, Wittgenstein talks about hinges (bedrock beliefs on which further discussion turns, but which are not open to falsification themselves), and draws parallelisms with mathematics that have become petrified. This way, mathematical petrification confers mathematical statements with a hinge-like status and is related to certainty and indubitability in a similar way as non-mathematical hinges like “there is a hand”: they are pivot points necessary to sustain epistemic practices (e.g., the counting of the fingers of my hand requires the hinge “there is a hand”, and apple transactions may depend on the hinge “2 + 2 = 4”).10Note that the literature on mathematical hinges lacks consensus on many fronts, like on their epistemic properties (Fairhurst, Pérez-Escobar and Sarikaya 2024; Kusch 2016; McGinn 1989; Moyal-Sharrock 2004) and even on whether there are mathematical hinges at all (Coliva 2020; Martin 2022). We do not aim to solve these open questions and our argument does not rely on their solution: that the later Wittgenstein’s philosophy of mathematics is related to his notion of hinges, the locality of many hinges (including mathematical hinges, or if there are no such hinges, including hinge-like mathematical propositions) and their basic role in disagreements among communities and individuals are not controversial. However, this does not mean that hinges are universal: in fact, most are specific to communities or sometimes even to specific people, thus accounting for disagreements about very concrete propositions. Therefore, the notion of hinge and Wittgenstein’s discussion of the properties of hinges are closely related to petrification in mathematics, but add something relevant for our aims: hinges often underlie specific epistemic practices, and not others. Accordingly, some propositions are immunised against doubt in some practices but not others because of different epistemic demands of the practices as a whole, leading to deep disagreements. In fact, there are deep disagreements in mathematics, even when this is one of the areas where most would not expect disagreements at all (cf. Aberdein 2023; Altenkirch 2023; Ernest 2023). Given our previous remarks on mathematical petrification and resilience to falsification, their similarity with hinges, that different hinges enable different epistemic practices, and the fact that against our common intuition of mathematics we do find disagreements in this discipline, suggest that petrification is typically a local phenomenon, leading to local mathematical hinges underlying disagreements. Mathematics can petrify locally, that is, some mathematical *practices* are carried out and *experienced* differently by different communities (some may not carry out or experience certain practices at all), leading to community-specific mathematical hinges on which further epistemic practices hinge. Although globalisation and the internet have made some of these processes less local by allowing more global practices and experiences, most work remains the endeavour of specific communities, research groups, and in some cases, single mathematicians. Therefore, a less idealised view of mathematical practices, where disagreements are conceivable, benefits from this integration of the later Wittgenstein’s notions of mathematical petrification (and its extended versions in secondary literature) and mathematical hinges. In this case, it allows us to understand how it is possible that Hamkins holds the view that CH is absolutely undecidable, while a sizable portion of the rest of mathematicians holds conflicting views: they constitute different epistemic practices regulated by different mathematical hinges, as a result of different experiences with mathematical practices and petrification processes over time. Other, vastly more popular views of mathematics, like formalism and platonism, would instead postulate that one party is wrong, and that the issue would be solved if this party checked their mathematical reasoning and found a mistake. This, however, does not seem to be an issue that can be solved in this way.

Now that we have shown how the notions of mathematical petrification and mathematical hinge are useful to understand Hamkins’ position on CH and existing disagreements, let us make explicit how it is related to the historicity of mathematics (although it is already implicit above): the process of petrification is a historical process inasmuch as it is the sustained and continuing use of models and experiences with them while obtaining systematic results in a given practice (by a community or individual) what leads to petrification in mathematics and the establishment of hinges. Therefore, the notion of petrification is not only useful to understand why Hamkins’ position on CH is the one it is, but also the historicity of mathematics leading to such a position and used by Hamkins himself to justify his position. Others have appealed to the historicity of mathematics to make sense of consensus and disagreement in this field (Wagner 2022); what we offer here is one mechanism by which such a relationship is brought to life.

But what does this tell us about petrification in contemporary set theory? First, we should note that these developments go beyond what set theory in its beginnings used to be. Cantor could not have thought about the intricate model constructions that developed out of the work of Gödel, Cohen, Solovay, Scott and others. Nowadays there are arguments that set theory shifted from a theory of the notion of set, to the study of models of set theory (cf. Antos 2024) and even the notion in question might have changed in reaction to the paradoxes and other foundational issues encountered (cf Boolos 1971 or Incurvati 2020). So far it has been established that there exist set theoretic models with different truth values for CH but also for many other set-theoretic statements. This is a shared hinge amongst some set theorists; see Koellner (2013) for disagreement. In this section we argue that there are still many parts of Hamkins’ argumentation that are not universally accepted among practitioners, i.e. which have petrified very locally, leading to local mathematical hinges. To explore this we sketch some set theoretic programs and see to what extent they fit to the narrative of Hamkins.

First, as argued by Rittberg (2016, 2024), the metaphysical views of practitioners may influence set theoretic practice, in the sense that these views may encourage certain lines of research, dissuade others, and drive what counts as relevant and important in the field.11This point can also be made outside of set theory, see f.i. (Carl et al. 2021; Fisseni et al. 2019; Fisseni, Sarikaya, and Schröder 2023), who stress the role of actual mental representation. Thus, underlying hinges in the form of metaphysical views may not be innocuous, not mere “philosophy” or “philosophical disagreements”, but mathematical disagreements. It is not contentious that beliefs about mathematics are historically contingent, like the question of which fields we consider to be more important than others, but this idea stresses how the window we have into the mathematical subject domain forms our beliefs on matters beyond proofs. The existence of non-standard models clearly triggers different potential interpretations. It is crucial that the monist would not deny Hamkins’ observations, but would argue that the models are in some way or other non-standard. Koellner (2013) argues that many constructed models are clearly non-standard. Say, the approach to use only countable transitive models, which are by construction set size, and more nuanced problems can be found for class size models. The most complicated cases to judge are perhaps in the context of the inner model program. Woodin (1999) developed complex approaches to settle the issue, like his Ω-logic idea which aims at distinguishing those models that are non-standard, but this inquiry never came to the envisioned conclusion (Rittberg 2015).

Some practices that accepted the metaphysical view are the modal logic of forcing (cf. Hamkins and Löwe 2008; Rittberg 2010), where the main idea is to study how set-theoretic sentences behave under the different forcing constructions.12It is of course not necessary to subscribe to the metaphysical view to contribute to this stream of research, see (Rittberg 2016, 2024) Recall that CH is a switch. So I can start with a model where it holds, then force an extension where it is false, then I can force one where it holds, etc. The truth-values of other set-theoretic statements cannot be changed once they have been fixed by a forcing extension, e.g. models in which V = L fails have no forcing extension in which V = L holds. Sentences like “V does not equal L” are buttons: once they are pushed, they cannot be unpushed (by means of forcing). Hamkins and Löwe (2008) already observed that “in fact every statement in set theory is *either a button, the negation of a button, or a switch*”. Another program that fits to Hamkins’ views is *Set-Theoretic Geology* (cf. Fuchs, Hamkins and Reitz 2015)*.* Here the direction is reversed, rather than constructing extensions the program looks at what different starting points could have looked like to generate a given model (which is naturally motivated by multiversism, whereas monism lacks such strong intrinsic motivation). In other words, this mathematical program looks for possible grounds (cf. Hamkins and Löwe 2013).

Hamkins’ modal logic of forcing and set-theoretic geology programs, which treat all set-theoretic models as metaphysical equals, are not mainstream programs in set-theoretic practice. One influential other program that is not committed to metaphysical equality amongst all different set-theoretic models is the study of the generic multiverse. Scholars pick one concrete model and then regard all forcing extensions of that model (cf. Woodin 2011). That is, in the generic multiverse only forcing extensions of a ground model are considered, whereas Hamkins considers *all* set-theoretic models, regardless of how they were constructed. Steel would look at the generic Multiverse as a formal first order theory. The Hyperuniverse program studies the collection of all countable transitive models of ZFC (cf. Arrigoni and Friedman 2013), so the focus is on a very particular kind of model that is technically well behaved. Here we should note that we in some sense misrepresent the position, as they all could still study different structures with different goals, we do not describe the full mathematical agenda but just a study of concrete objects in question. Also some opinions change: e.g. Woodin famously changed his mind (cf. Rittberg 2015).

Even forcing is not as unproblematic as it might sound. Hamkins (2015) says “Although it was formerly common to undertake forcing constructions only for countable transitive models of fragments of ZFC, one may formalise the forcing method as an internal ZFC construction, rather than a metatheoretic construction, and thereby make sense of forcing over an arbitrary model of set theory, including over the full universe V.” But once again not all set theorists agree on this.

It remains to see if the many different set theoretic practices will lead to alternative petrifications (and therefore, local mathematical hinges) but it seems clear that all set theorists wish to continue to do forcing constructions. But communities and practitioners disagree about the metaphysical weight of the set-theoretic models involved and this leads to different set theoretic endeavours (with potentially even more different actual goals behind these endeavours, as each practice could be done for the sake of itself or as a way of contributing to another goal).

## 6 Does Other Mathematics Petrify? Parallelisms Under Petrification

The case of CH seems to be an extraordinarily interesting one, but we should note that Hamkins himself makes an interesting parallel to the case of non-euclidean geometry. The experience we make with such non-euclidean models contributed to their acceptance. Hamkins makes this parallel to further clarify his position on the decidability of CH:

In time, however, geometers gained experience in the alternative geometries, developing intuitions about what it is like to live in them, and gradually they accepted the alternatives as geometrically meaningful. (Hamkins 2012, pp. 425–426)

He continues

A stubborn geometer might insist – like an exotic-travelogue writer who never actually ventures west of seventh avenue – that only Euclidean geometry is real and that all the various non-Euclidean geometries are merely curious simulations within it. Such a position is self-consistent, although stifling, for it appears to miss out on the geometrical insights that can arise from the other modes of reasoning. Similarly, a set theorist with the universe view can insist on an absolute background universe V, regarding all forcing extensions and other models as curious complex simulations within it. (I have personally witnessed the necessary contortions for class forcing.) Such a perspective may be entirely self-consistent, and I am not arguing that the universe view is incoherent, but rather, my point is that if one regards all outer models of the universe as merely simulated inside it via complex formalisms, one may miss out on insights that could arise from the simpler philosophical attitude taking them as fully real. (Hamkins 2012, p. 426)

Koellner (2013) argues against the parallelism that Hamkins draws between set theory and geometry. Koellner makes a distinction between *formal mathematics* and *concrete mathematics*. Formal mathematics aims at discerning structural features that pertain to diverse mathematical structures. Koellner gives group theory, ring theory, and topology as examples. In formal mathematics, the axioms characterise the class of structures one is interested in, claims Koellner. Since formal mathematics is not concerned with a single structure, it is not the aim of formal mathematics to settle whether a certain axiom holds for the class of structures under consideration. “There has never, for example, been a program to settle the axiom of commutativity” (p.13). Concrete mathematics on the other hand aims at establishing which axioms hold for a single structure (up to isomorphism). Koellner gives arithmetic, analysis, and, importantly, set theory as examples.

In this terminology, Hamkins argues that considering the experiences set theorists have with the various set-theoretic models, set theory should no longer be thought of as a case of concrete mathematics. Rather, set theory is formal mathematics (according to Hamkins in Koellner’s terminology). Koellner provides numerous arguments against this. Hamkins’ parallelism between set theory and geometry is treated in a dedicated section.

According to Koellner, geometry is not easily situated as either concrete nor formal mathematics. In its beginnings, Koellner claims, geometry was conceived as having elements of both: it was concrete in that it tracked features of physical space, but it was also formal in that it was concerned with an idealised space. Yet things changed, so Koellner claims, after the independence of the parallel postulate became known. “What is clear is that after Riemann the parallel postulate clearly lost its significance as an independent question in the context of formal geometry. But still, there was a question that remained; perhaps not a question that is equivalent to the old question, but one which nonetheless remained - was physical space Euclidean” (p.17). (The answer to this last question is ‘no’.)

According to Koellner, Hamkins has not given us sufficient reason to think that set theory is like geometry. It is certainly different from physical geometry. And that it is like formal geometry is just like claiming that set theory is like any other branch of formal mathematics. Such arguments, Koellner maintains, have “no traction with the opponent” (p.12).

In spite of Koellner’s reservations regarding Hamkins’ parallelism between CH and geometry, we argue that there is a new parallelism to be made based on the notion of mathematical petrification that we unpacked in the preceding section. But first, there is a difference: geometry has applications. Thus, Euclidean geometry petrifies in the most common Wittgensteinian sense: because of repeated experience with it and its direct applications. The empirical starting points are, especially, stretched ropes in Ancient Greece and Ancient Egypt; see (Asper 2009). Ropes are stretched in certain ways to measure land area and the same results are obtained again and again, validated by systematic *experience* with this *practice*. Of course, the empirical starting point is not formal: when rope stretchers drop the ropes, the shapes they formed vanish. But those shapes become mathematically petrified into shapes like triangles, squares together with their extended formal properties. For instance, “the angles of a triangle amount to 180°” is a mathematical hinge emerging from a petrification of empirical regularities and our experiences stretching ropes and drawing and measuring triangles, and dubitability is impractical in this *practice*.

However, a different mathematics emerged in certain contexts. While Euclidean geometry remains petrified in its domain of applicability, non-Euclidian geometry is applicable in contexts like cartography and Einstein’s theory of relativity. Gauss found that one of Euclid’s five postulates, the parallel postulate, did not hold for curved surfaces.13This paper simplifies historical developments starting earlier and the contributions of others like Lobachevsky and Bolyai are not adequately represented. In the context where Euclidean geometry petrified, surfaces were not curved: ropes were stretched on thin air by land surveyors and lines were drawn on flat surfaces. Euclidean geometry never petrified in the context of curved surfaces, and thus we have two parallel petrification processes, so that two geometries can coexist. Taken as a whole, the notion that we do not have to choose between one geometry or the other petrifying in the sense that we have two petrifications and we cannot erase what has been successfully practised just because we need to choose “the” one true geometry. For instance, we could conclude that Euclidean geometry is wrong, but at a practical level, the practices worked! The fact that some surfaces were problematic for Euclidean geometry, as revealed by Gauss, does not erase past successful practices.

Overall, this qualifies as a Wittgensteinian reconstruction of Hamkins’ position on Euclidean and non-Euclidian geometries, and by extension, his position on the absolute undecidability of CH. What we get is that both Euclidean and non-Euclidian geometries having a place in mathematics became petrified, and so did CH and ¬CH, and, importantly, that what could otherwise be considered “structural flaws” of mathematics are dealt with tolerance in several contexts. This is related to another important feature of Wittgenstein’s later philosophy of mathematics that makes us understand tolerance to undecidability: for Wittgenstein, problems and inconsistencies eventually manifested in mathematics, like “hidden contradictions” of which we were not aware, are not real problems of mathematical practices, for two reasons: 1) the manifestation of the contradiction cannot change the past, for instance how successful practices were, and 2) practices can find ways to work around contradictions in both natural language and mathematics. Of course, this sounds radical to most people, including Turing, who attended Wittgenstein’s lectures in Cambridge. However, this is itself consistent with Wittgenstein’s overall view of mathematics: basically, viewing mathematics as rule-following practices and departing from the structural analogies underlying platonism and formalism, makes things like contradictions more acceptable (as long as it does not disrupt our mathematical practice; i.e., as long as, given a certain mathematical training or else, our rule-following is not disrupted by the contradiction). E.g., one could just “not derive anything from the contradiction”, or apply inconsistent mathematics in certain successful ways. The point is to find ways to follow rules explicitly expressed as contradictions or featuring “hidden contradictions” so that things go as expected without disrupting our practices. In fact, it is well documented that inconsistent mathematics remain applicable without explosions, like Dirac’s delta function in physics (see Pérez-Escobar and Sarikaya 2022, for more details on this). And, lastly, undecidability (“we should not decide”) is tolerated in the case of Hamkins, and even “petrified” given the historicity of previous practices, at the expense of other structural considerations (like the desideratum that mathematical propositions should be decidable). Thus, overall, Wittgenstein’s philosophy accounts for general structural tolerance as a principle that explains more than just tolerance of absolute undecidability.

Note that this is not exactly the point that Hamkins makes: he rather stresses an inner mathematical perspective of actual experience with the non-Euclidean models in his parallelism between geometry and set theory. However, it is precisely the point that Hamkins stresses which, according to our points in this work, account for the petrification of mathematics without direct applications! Namely, in Wittgensteinian terms, mathematical rules have been followed in certain ways (e.g., conducting some mathematical derivations and not others) by some communities with similar training in certain contexts over time, the results of which are integrated into their training in a feedback loop, leading to regularities of their own kind. Before this process, however, for Hamkins (at least for his view on the multiverse) and according to our framework, there was no “right” and “wrong”; recall his claim that “As a result [of the set theorists’ experiences with CH and ¬CH models], I argue, the continuum hypothesis can *no longer* be settled in the manner formerly hoped for” (our emphasis). These regularities-that-become-regulative are common to mathematics with and without direct applications, and so, under this perspective, the distinction that geometry does have applications is not too significant.

## 7 Conclusions

In this paper we provided a Wittgensteinian engagement with the set-theoretic pluralism debate in terms of petrification, i.e. the process through which successful ways of doing things generate normative demands. We focussed specifically on Hamkins’ radically pluralist position. In the terminology of this paper, Hamkins argues that our experiences with various set-theoretic models in which CH holds and those in which CH fails have petrified into the absolute undecidability of CH. We have pointed out that not all set theorists agree, and have phrased this in terms of local hinges: for radical pluralists like Hamkins these experiences have petrified into some normative force, for other set theorists they have not. We pointed out that Hamkins’ argument is in particular entangled with claims about the metaphysical equality of all set-theoretic models involved. This metaphysical equality claim is rejected by some set theorists. We made the further case that the practice, in its understanding as ‘way of doing things’ without further interpretation, of building various set-theoretic models has petrified into the normative claim that set theory ought to be such that these models can be built and studied. This aspect, we suggested, has normative force for the set-theoretic community at large, i.e. it is a global hinge. That is, Hamkins has it right that the set theorists’ experiences with building various set-theoretic models have petrified into a hinge, but the hinge he suggests (‘absolute undecidability of CH’) is ‘merely’ a local hinge. The global hinge is ‘set theory ought to be such that these models can be built’. Experiences thus shape the hinges of mathematics, which is one more dimension of the historical contingency of mathematics.
